# Sensitivity of initial biopsy or transurethral resection of bladder tumor(s) for detecting histological variants on radical cystectomy

**DOI:** 10.1186/s12894-015-0037-2

**Published:** 2015-05-30

**Authors:** Peng Ge, Zi-Cheng Wang, Xi Yu, Jian Lin, Qun He

**Affiliations:** Department of Urology, Peking University First Hospital and Institute of Urology, Peking University, Beijing, 100034 China; National Research Center for Genitourinary Oncology, Beijing, 100034 China

**Keywords:** Urothelial carcinoma, Variant histologic differentiation, Cystectomy, Pathology, Prognosis

## Abstract

**Background:**

To investigate the efficacy of initial biopsy or transurethral resection of bladder tumor for detecting histological variants on radical cystectomy and to assess the prognostic significance of variant histology on urothelial carcinoma outcomes after radical cystectomy.

**Methods:**

Clinical and histopathological characteristics of 147 patients with variant histology who underwent radical cystectomy for urothelial carcinoma between 2006 and 2012 were assessed. Sensitivity was calculated as the proportion of radical cystectomy specimens with a particular variant that also presented the variant in the biopsy or transurethral resection specimen. The Kaplan-Meier method and multivariate Cox proportional hazard regression analysis were used to estimate cancer-specific survival.

**Results:**

Of the 147 patients, 116 (79 %) were diagnosed with a single variant histology, and 31 (21 %) had multiple patterns. Squamous differentiation (31 %) was the most common single variant histology, followed by glandular differentiation (28 %). Except for small cell variant (100 %), the sensitivity of biopsy and transurethral resection was most effective for the diagnosis of squamous differentiation, 19 % vs. 40 % respectively, followed by glandular differentiation, 11 % vs. 21 % respectively. A total of 6 % and 49 % patients could be variant-free partially due to biopsy or complete resection(s) respectively. Presence of variant differentiation in urothelial carcinoma at cystectomy was significantly associated with inferior survival both in univariate analysis (*P* = 0.005) and multivariate analysis (HR4.48, 95 % CI:1.03-19.53).

**Conclusions:**

Overall sensitivity of biopsy or transurethral resection to detect variant differentiation on cystectomy is relatively low. Patients with variant differentiation on cystectomy specimens have inferior survival.

## Background

More than 90 % of bladder cancers are urothelial carcinomas. Urothelial carcinoma of the bladder (UCB) has a propensity to undergo divergent or variant differentiation, resulting in a wide spectrum of subtypes [1–3]. Thirteen morphotypes were discussed in the 2004 *World Health Organization Classification of Tumours of the Urinary Tract* [4]. Since then, several new divergent subtypes have been described [2, 5].

Generally, about 7 to 81 % of bladder UCBs have some type of variant differentiation [3, 6–8]. The recognition of histological variants in UCB is of significance to both pathologists and clinicians because (a) some variants may affect prognostic consequences, (b) some may need different modalities compared with those used in conventional UCB, and (c) knowledge of the histological variants may be crucial to avoid diagnostic misinterpretations [5]. That applies, for example, to tumors consisting of plasmocytoid or micropapillary components. UCB patients with plasmocytoid histology may lead to diagnosis delays and inappropriate therapies because of absence of gross hematuria and the lack of visible tumor at cystoscopy [9, 10]. Given that micropapillary carcinoma is less sensitive to immunotherapy or chemotherapy compared to traditional UCB, one leading group has suggested early cystectomy should be taken into consideration in the clinical management of those patients with micropapillary histology for pTa and pT1 tumors [5, 11]. Hence, early detection of variant differentiation is crucial.

However, the diagnosis of UCB variants may be challenging. A common feature of the variant patterns is that the frequency with which they are diagnosed is influenced by many factors: the extensiveness of the tumor pathologically sampled, the proportion of the divergent element of the whole tumor, the attentiveness of the pathologist detecting small foci of the respective pattern, the severity of artifact caused by tangential sectioning, cautery and mechanical injury, etc. [3–5, 12].

This study was aimed at investigating the sensitivity of biopsy and/or transurethral resection of bladder tumor (TURBT) for detecting the histological differentiation on radical cystectomy (RC). We also assess the prognostic significance of variant histology in UCB at cystectomy.

## Methods

### Patient population and pathologic evaluation

This was a retrospective, single-institution study approved by Peking University First Hospital review board (No. 2014692). We retrospectively reviewed all the pathology reports available at our institution with informed consent for each UCB patient treated with RC from the time of first visit to the time of RC. The variant histology in this study are those recognized by 2004 WHO Classification of Tumors and the literature [4, 5]. Four highly experienced pathologists specializing in urologic pathology were involved in reviewing these cases. Pathological grade and stage were assigned according to the recommendations, respectively [4, 13]. Any problematic cases were re-reviewed by a dedicated urologic pathologist (author QH) to verify the initial diagnoses. Any amount of variant differentiation was reported.

Inclusion criteria: (a) From the time of first visit to the time of RC, all the treatments were done at our hospital; first visit was defined as patients had never received biopsy or underwent biopsy for only once at other institutions before coming to our hospital and the pathological sections of biopsy were re-reviewed by the pathologists at our medical center; (b) Detailed medical record information was available. Exclusion criteria: (a) Patients received TURBT(s) or partial cystectomy at other institutions before initiation of therapy at our institution; (b) Patients presented extravesical malignant primary tumors before RC; (c) Patients underwent biopsy or TURBT without subsequent RC as well as any with pure nonurothelial morphology [1].

Between 2006 and 2012, a total of 620 consecutive patients were treated with RC for UCB at Peking University First Hospital. Ultimately, 147 patients who met all the above-described criteria with a diagnosis of variant differentiation in pure or mixed form after RC, the matched preceding biopsy or TURBT(s), were included in this study. Among those patients, 63 who underwent biopsy only once without TURBT were included in the analysis of biopsy sensitivity, while the remaining 84 who underwent TURBT at least once were used to calculate the sensitivity of TURBT. For study purposes TURBT was defined as resection of all visible tumor while biopsy partial sampling [1].

### Follow-up

Follow-up was performed generally quarterly for the first year, semiannually for the next 2 years and annually thereafter with laboratory and imaging studies unless otherwise clinically indicated. Outcomes of interest was cancer-specific survival (CSS). CSS duration was calculated from date of cystectomy to death due to bladder cancer; surviving patients were censored at last follow-up [14, 15].

### Statistical analysis

Sensitivity was calculated as the proportion of RC specimens with a particular variant that also had the variant in the biopsy or last TURBT (LTURBT) specimen [1]. The Kaplan–Meier method was used to graphically display survivor functions. Multivariate Cox proportional hazards models were used to estimate independent relationships between categorical variables that were univariably prognostic for CSS. The assumptions of proportional hazards with respect to the log-hazard were checked using the Schoenfeld residual test and no major model violations were observed. All analyses were performed using SAS version 9.2 (SAS Institute, Cary, NC, USA) and/or SPSS version 20.0 (IBM Corp, Armonk, NY, USA). Two-sided *P* ≤ 0.050 was considered statistically significant.

## Results

### Histologic spectrum of variant differentiation

Of the 147 patients, 116 (79 %) were diagnosed with a single variant histology, whereas the remaining 31 (21 %) had multiple patterns of variant histologic components on RC, the matched preceding biopsy or TURBT(s). In decreasing order of frequency, the spectrum of divergent differentiation included: squamous (31 %), glandular (28 %), sarcomatoid (12 %), small cell (2 %), clear cell (2 %), microcystic (2 %), lymphoepithelioma-like (1 %), and undifferentiated (1 %).

### Sensitivity of biopsy or TURBT for detecting histological variants on RC

The sensitivity of biopsy and LTURBT to detect a certain variant varied (Table 1). Except for small cell variant (100 %), the sensitivity of biopsy and LTURBT was most effective for the diagnosis of squamous differentiation, 19 % vs. 40 % respectively, followed by glandular differentiation, 11 % vs. 21 % respectively. Sarcomatoid variant with a sensitivity of 9 % was only detected on LTURBT. Surprisingly, it seemed to be invalid for biopsy or LTURBT to detect lymphoepithelioma-like (0 %), plasmacytoid (0 %), clear cell (0 %), nested (0 %), and undifferentiated variant (0 %).

**Table 1:** The sensitivity of biopsy and LTURBT for detecting variant differentiation

Variant	No. biopsy	RC	% sensitivity	No. LTURBT^a^	RC	% sensitivity
Squamous	8	28	19	26	20	40
Glandular	4	27	11	18	19	21
Sarcomatoid	0	19	0	14	11	9
Small cell	1	1	100	1	3	0
Lymphoepithelioma-like	0	3	0	0	0	0
Plasmacytoid	0	1	0	0	1	0
Clear cell	0	2	0	0	3	0
Nested	0	0	0	1	0	0
Microcystic	0	3	0	1	1	0
Undifferentiated	0	1	0	0	0	0

^a^Last transurethral resection of bladder tumor precystectomy

Forty-five patients (31 %) exhibited variant(s) only on biopsy or TURBT(s), including 4 cases for biopsy, and 41 cases for TURBT(s). In other words, this suggested that the efficacy of biopsy and TURBT(s) upon removing the variant components during the disease course was 6 % (4 of 64) and 49 % (41 of 84) respectively partially due to extensive sampling and complete resection. There were 71 patients who underwent TURBT only once and 13 underwent TURBT at least twice (varying from twice to six times) during the time of first visit to the time of RC. For those who underwent TURBT only once, 34 (48 %) were found free of variant complements on RC. Interestingly, for patients who underwent TURBT at least twice, 5 patients were diagnosed with different variant histologic subtypes on RC, the matched preceding biopsy or TURBT (Table 2).

**Table 2:** Variants detected on TURBT and RC of patients who underwent TURBT at least twice (n = 13)

Patient ID	No. TURBT^a^	No. variant^b^	RC^c^	Variant (TURBT/No.TURBT)^d^
1	6	1	Glandular	Sarcomatoid (6/6)
2	5	1	UC^e^	Clear cell (1/5)
3	4	1	Glandular	Glandular (4/4)
4	4	2	UC	Squamous (2/4), Nested (4/4)
5	3	1	Sarcomatoid	Glandular (3/3)
6	2	1	Glandular	Squamous (2/2)
7	2	1	Squamous	Squamous (2/2)
8	2	2	Squamous	Squamous (1/2), Squamous (2/2)
9	2	1	UC	Squamous (1/2)
10	2	1	UC	Small cell (2/2)
11	2	1	UC	Squamous (1/2)
12	2	1	UC	Sarcomatoid (1/2)
13	2	1	UC	Squamous (1/2)

All the specimens were re-reviewed by a dedicated pathologist QH

^a^The total number of TURBT

^b^The total number of TURBT with a diagnosis of variant differentiation

^c^The diagnosis of radical cystectomy specimen

^d^TURBT/No.TURBT = The ordinal number of TURBT with a diagnosis of variant differentiation / The total number of TURBT

^e^Urothelial carcinoma without variant differentiation

### Cancer-specific survival

**Fig. 1:**
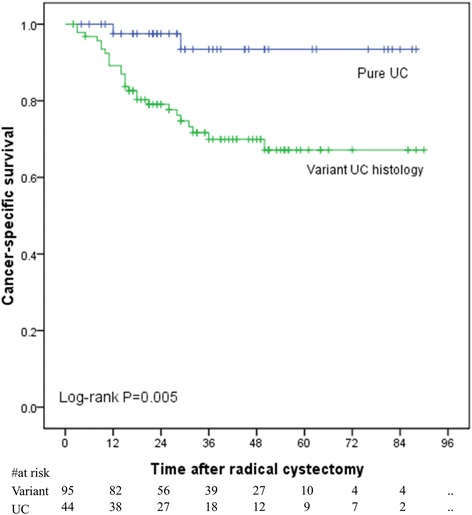
Kaplan-Meier plot displays estimated cancer-specific survival in 139 patients treated with radical cystectomy, stratified by pure vs. variant differentiation in urothelial carcinoma at cystectomy (UC, urothelial carcinoma)

Follow-up information was available for 139 patients (94.6 %); 28 patients died of bladder cancer during follow-up. The mean age of the 139 patients was 66 years (SD:11.1). The median follow-up was 31 months (range 2–90). Of those, 95 UCB patients were identified with variant differentiation on RC specimens. Fig. 1 shows that variant differentiation on RC was statistically significantly associated with inferior survival (*P* = 0.005). Similarly, multivariate analyses after being adjusted for the effects of pathologic stage demonstrated that presence of variant differentiation in urothelial carcinoma at cystectomy was independently associated with cancer-specific mortality (HR4.48, 95 % CI:1.03-19.53).

## Discussion

In this current study, we investigated the sensitivity of biopsy and/or TURBT for detecting the histological differentiation on RC and assessed the prognostic significance of variant histology on urothelial carcinoma outcomes after RC. Our results indicate that overall sensitivity of biopsy or TURBT to detect variant differentiation on RC is relatively low. Presence of variant differentiation in urothelial carcinoma at cystectomy portends inferior survival.

It is not an uncommon phenomenon that UCB has a great propensity to undergo variant differentiation. In accordance with previous studies [3, 15], the most prevalent mixed differentiation in this current series was squamous, accounting for 31 % of all cases. Squamous differentiation, characterized by the presence of intercellular bridges, keratin pearls or keratinization, has an unfavorable response to radiation and chemotherapy compared with pure UCB [5]. Previous studies have shown that its frequency runs parallel with grade and stage [16, 17]. Glandular differentiation, as the second common variant, is exemplified by the presence of true glandular spaces within the tumor, presented in 28 % of all cases [4]. After squamous and glandular subtypes, there is sarcomatoid differentiation, which is featured by biphasic malignant neoplasms with the evidence of expressing epithelial and mesenchymal markers, accounting for 12 % of all cases. In a sarcomatoid carcinoma, molecular evidence strongly argues for a monoclonal origin of both epithelial and mesenchymal components [18, 19]. Additionally, history of previous radiation or cyclophosphamide can be a valuable clue in ascertaining the diagnosis [4]. Other unusual architectural patterns (small cell, lymphoepithelioma-like, clear cell, microcystic, undifferentiated) of UCB only accounted for a little proportion of the whole, namely 8 %. Those variants, compared with squamous and glandular differentiation, are more complicated for the pathologist to reach a direct diagnosis and can sometimes mimic reactive processes, benign lesions or metastasis of other tumors. For instance, lymphoepithelioma-like carcinoma, for which the sine que non feature is the presence of a pronounced lymphoid infiltrate, may be partially or substantially obscured by a variable desmoplastic response, a brisk inflammatory infiltrate and even a prominent chronic inflammatory cell infiltrate [5].

Although some studies have contributed to raising the awareness of these entities and improved diagnosis, the diagnosis of UCB variants may still be challenging for (a) the limitation of biopsy samples and transurethral resectates available to the pathologist, (b) the artifact caused by tangential sectioning, cautery and mechanical injury, and (c) even more importantly the difficulty of discriminating over selected mimics [3–5, 12]. In this study, the most sensitive variants detected by biopsy and LTURBT are squamous and glandular subtypes. This finding corroborates a previous study in which Ahmed et al. reported that the sensitivity of TURBT to detect squamous and glandular variant was 53 % and 25 % respectively [1]. Similar to the squamous differentiation, the sensitivity of LTURBT to detect glandular variant is nearly twice as that of biopsy. Compared to other variants, the sensitivity of biopsy and LTURBT to detect squamous and glandular differentiation was relatively higher. For most variants, Table 1 suggests an unfavorable sensitivity. However, considering their nature of rarity, more studies based on multicenter and/or international collaboration with large sample size are warranted to further validate these conclusions.

It is well known that sensitivity lies in correct diagnoses. In order to make a correct diagnosis, several points should be noted. First, recognition of the immunochemical profile is quite important for pathologists to distinguish certain variants from the confounding variables. Mahul et al. [5] summarized the immunohistochemical markers associated with urothelial differentiation in detail. Second, morphology should be made the best advantage. One case in point is that immunohistochemistry is less significant compared with morphology in discrimination nested variant over florid von Brunn’s nests, as the reliable immunohistochemical cut-point is difficult to be determined [12]. Besides, in some cases, clinicopathological correlation is helpful in excluding an extravesical primary tumor [5]. Recently, Hughes et al. [20] have reported the effect of Fourier transform infrared (FTIR) microspectroscopy to diagnose some selected variants, which might open a new door for variant diagnosis.

In this study, we found that 49 % patients who underwent TURBT at least once were not diagnosed with variants on RC specimens. That is to say, approximately 50 % of the patients could be in variant-free condition partially due to complete resection(s). Dissimilarly, Ahmed et al. reported that 6 % (9/159) patients demonstrated variant(s) only on precystectomy biopsy or TURBT [1]. The factors that could influence the variant diagnosis could also explain the discrepant results between the two studies. Interestingly, among the 13 patients who underwent TURBT at least twice, 12 of them presented variants detected in some but not in all TURBTs (Table 2). Similarly, Matthew et al. found that some patients had a different or additional mixed histologic type on cystectomy than they did on transurethral resection of bladder tumor [21]. This raises several practical questions encountered in clinical work. First, for those who are diagnosed with different variant differentiation at biopsy, TURBT(s) or RC, what treatment algorithms should be applied in clinical management? Besides, for those who are diagnosed with variant differentiation at biopsy/TURBT and without variant subtype diagnosis on RC, should we adopt the same follow-up strategies used on the UCB patients without a diagnosis of variant differentiation during the disease course? Further investigations are needed to validate the paradigms.

Despite a number of individual studies have evaluated the impact of histological variants on prognosis of patients with UCB, this question remains in debate [14, 22]. We also evaluated the impact of variant differentiation on clinical outcomes. Of the 139 patients with available follow-up information, 95 UCB patients were identified with variant differentiation on RC specimens. Patients with variant differentiation on RC specimen have inferior survival both in univariate analysis (*P* = 0.005) and multivariate analysis (HR4.48, 95 % CI:1.03–19.53). Of note, our data were limited by an overall shorter median follow-up time of 31 months and small sample size. The statistical power was weakened by very few recurrent and death events. More investigations with a large number of patients, with a longer follow-up time and with a centralized pathologic re-review are warranted to validate these conclusions. Ultimately, our understanding of urinary bladder cancer biology should not be limited to histologic variants. The underlying genetic and molecular drivers of tumor induction, promotion, and progression and as well as makers of chemosensitivity also need to be investigated [14].

Our study is not devoid of potential limitations that need to be addressed. First and foremost are the limitations inherent to its retrospective nature. Besides, the follow-up time and sample size were the limitations of this study.

## Conclusions

Overall sensitivity of biopsy or TURBT to detect variant differentiation on RC was relatively low. Nearly 50 % of the patients could be variant free partially due to complete resection(s). Patients with variant differentiation on RC specimen have inferior survival. Further studies on the approach to early detection of variant differentiation and the management tactics for the UCB patient presenting different variant differentiation at biopsy, TURBT or RC, may be facilitated.

## Abbreviations

UCB: Urothelial carcinoma of the bladder
TURBT: Transurethral resection of bladder tumor
RC: Radical cystectomy
CSS: Cancer-specific survival
LTURBT: Last transurethral resection of bladder tumor
